# Using linked cohort data to help address residual confounding in analyses of population administrative data

**DOI:** 10.23889/ijpds.v9i5.2544

**Published:** 2024-09-18

**Authors:** Richard Silverwood, Gergo Baranyi, Lisa Calderwood, Bianca De Stavola, George Ploubidis, Katie Harron

**Affiliations:** 1 University College London

## Objectives

Analyses of population administrative data can often only be minimally adjusted due to the unavailability of a full set of control variables, leading to bias due to residual confounding. Cohort studies will often contain rich information on potential confounders but may not be sufficiently powered to meaningfully address the research question of interest. We aimed to use linked cohort data to help address residual confounding in analyses of population administrative data.

## Approach

We propose a multiple imputation-based approach, introduced through application to simulated data. We then apply this approach to a real-world problem – examining the association between pupil mobility (changing schools at non-standard times) and Key Stage 2 attainment using data from the UK National Pupil Database (NPD). The limited control variables available in the NPD are supplemented by multiple measures of socioeconomic deprivation captured in linked Millennium Cohort Study (MCS) data.

## Results

The proposed approach is observed to perform well when using simulated data. The association between pupil mobility and Key Stage 2 attainment was attenuated after supplementing the NPD analysis with information from linked MCS data, though with a decrease in precision.

## Conclusion

We have demonstrated the potential of the proposed approach, but more work is required.

## Implications

The principles underlying this innovative approach are widely applicable: any analysis of administrative data where confounder control is limited by the availability of information could potentially be strengthened by linking a subset of individuals into richer cohort data and leveraging the additional information to inform population-level analyses.

